# Nicotinamide attenuates streptozotocin-induced diabetes complications and increases survival rate in rats: role of autonomic nervous system

**DOI:** 10.1186/s12902-021-00795-6

**Published:** 2021-06-28

**Authors:** Paula L. Cruz, Ivana C. Moraes-Silva, Amanda A. Ribeiro, Jacqueline F. Machi, Marcelo Dantas Tavares de Melo, Fernando dos Santos, Maikon Barbosa da Silva, Celia Maria Cassaro Strunz, Elia Garcia Caldini, Maria-Claudia Irigoyen

**Affiliations:** 1 Heart Institute (InCor), University of São Paulo Medical School, Av. Dr. Enéas de Carvalho Aguiar, 44 - Bloco 1, subsolo, São Paulo, SP 05403-900 Brazil; 2 Department of Pathology, University of São Paulo Medical School, Sao Paulo, Brazil

**Keywords:** Nicotinamide, Diabetes, Autonomic nervous system

## Abstract

**Background:**

To evaluate the effect of nicotinamide prior to streptozotocin-induced (STZ) diabetes in baroreflex sensitivity and cardiovascular autonomic modulation, and its association with hemodynamics and metabolic parameters.

**Methods:**

Methods: Male Wistar rats were divided into control (Cont) and STZ-induced diabetes (Diab). Half of the rats from each group received a single dose of nicotinamide (100 mg/Kg) before STZ injection (Cont+NicA and Diab+NicA). All groups were followed-up for 5 weeks.

**Results:**

Body weight loss of more than 40% was observed in Diab throughout the period (Diab: 271.00 ± 12.74 g; Diab+NicA: 344.62 ± 17.82). Increased glycemia was seen in Diab rats (541.28 ± 18.68 mg/dl) while Diab+NicA group had a slight decrease (440.87 ± 20.96 mg/dl). However, insulin resistance was observed only in Diab. In relation to Cont, heart rate, mean blood pressure and diastolic function were reduced when compared to Diab, together with parasympathetic modulation and baroreflex sensitivity. All of these parameters were improved in Diab+NicA when compared to Diab. Improved baroreflex sensitivity and parasympathetic modulation were correlated with glycemia, insulin resistance, and body weight mass. Additionally, Diab+NicA group increased survival rate.

**Conclusions:**

Results suggest that the association of nicotinamide in STZ-induced diabetic rats prevents most of the expected derangements mainly by preserving parasympathetic and baroreflex parameters.

## Introduction

Diabetes mellitus (DM) is a complex metabolic disease affecting about 422 million people worldwide. It is characterized by chronic hyperglycemia, insulin resistance, and/or insulin secondary deficiency caused by the failure of β-pancreatic cells [1, 2].

According to the classification proposed by the American Diabetes Association (ADA), there are two DM types: type 1 diabetes and type 2 diabetes are heterogeneous diseases in which clinical presentation and disease progression may vary considerably [1]. Type 1 diabetes is usually diagnosed in children and adolescents. It is an autoimmune disease characterized by the destruction of β-pancreatic cells by lymphocytes and macrophages resulting in hyperglycemia, usually leading to absolute insulin deficiency [3]. Type 2 diabetes, which accounts for 90–95% of all cases of DM, occurs due to a progressive loss of β-cell insulin secretion frequently on the background of insulin resistance compensated by increased secretion of insulin [1, 2]. However, this prolonged overstimulation of insulin secretion leads to progressive exhaustion and degradation of β-cells [4, 5]. Multiple risk factors for type 2 DM are associated with bad eating habits, increased adiposity and sedentarism, leading to the hypothesis that type II DM is a result of gene-environment interactions [6, 7].

Different experimental models emerged as possibilities to mimic human types 1 and 2 DM to study its development and complications. Masiello et al. (1998) developed a rat model of diabetes induced by administration of streptozotocin (STZ) and nicotinamide. STZ selectively destroys pancreatic beta cells, while N decrease the damage caused by STZ, creating a state of partial insulin deficiency, similar to what occurs in type 2 diabetes. The severity of STZ + N-induced diabetes is much lower than that of diabetes induced by STZ alone; rats manifest moderate hyperglycemia and do not require exogenous insulin to survive [8]. Type 2 DM is probably to be as elaborate and heterogeneous as the human condition. Thus, in some animals, insulin resistance predominates, while in others there are predominance of β-cell failure. Models where glucose intolerance is part of a large phenotype, such as: obesity, dyslipidaemia and hypertension may also provide valuable insights into human type 2 DM [9].

Nicotinamide, a derivative of vitamin B3, effectively protects β-cells against the cytotoxicity of STZ. The protective action of N relies on the decreased damage and deoxyribonucleic acid (DNA) methylation in pancreatic islets, improved insulin secretion [10, 11], neuroprotection and antioxidant functions [12].

Although all of these mechanisms play a pivotal role in diabetes development, autonomic nervous system also underlies cardiometabolic pathophysiology in metabolic diseases. Studies in fructose-fed animals demonstrated that autonomic nervous system dysfunction, mainly represented by baroreflex sensitivity impairment, not only accompanies the disease progression but may also precede metabolic and hemodynamics alterations [13, 14]. Lin et al. (2008) showed early cardiac autonomic dysfunction and baroreflex impairment in diabetic rats pre-treated with nicotinamide; however, rats were anesthetized during the evaluation [15]. In young adults with diabetic parents compared to non-diabetic parents, autonomic alterations were observed despite differences in baseline glycemia levels [16].

Since autonomic control of circulation seems to modulate DM complications, and nicotinamide presents a protective role in diabetic rats induced by STZ, we hypothesized that preserved cardiovascular autonomic modulation and baroreflex sensitivity could also mediate the attenuation of STZ-induced derangements in rats pre-treated with nicotinamide. Therefore, this study aimed to evaluate the effect of nicotinamide prior to STZ-induced diabetes in baroreflex sensitivity and cardiovascular autonomic modulation, and its association with hemodynamics and metabolic parameters.

## Methods

### Animals and groups

This research was carried out in accordance with the ARRIVE procedures for animal experiments [17]. All experimental procedures were approved by the Ethics Committee for Animal Experimentation from the University of São Paulo Medical School (protocol no. 338/12). This investigation was conducted in accordance with the Guide for the Care and Use of Laboratory Animals published by the U.S. National Institutes of Health. Male Wistar rats (330–390 g) were housed in collective polycarbonate cages (four animals per cage) with a controlled temperature (22 °C) and a 12:12-h light-dark cycle. Rats were randomly divided into 4 four groups (6–8 rats/group): control (Cont), diabetic (Diab), control pre-treated with nicotinamide (Cont+NicA), and diabetic pre-treated with nicotinamide (Diab+NicA).

### Nicotinamide treatment and diabetes induction

On the day of diabetes induction, all rats underwent a 6-h fasting. Cont+NicA and Diab+NicA rats received a single intraperitoneal injection of 100 mg/kg of nicotinamide (Sigma-Aldrich) 15 min before diabetes induction [18, 19]. Diab and Diab+NicA rats were made diabetic by a single injection of freshly prepared STZ (50 mg/kg body weight) in citrate buffer (0.1 M, pH 4.5) in the tail vein [20]. Control animals received an intraperitoneal injection of NaCl 0.9% and citrate buffer without STZ. After 15 days of induction, glycemia was measured and only animals with levels higher than 180 mg/dl were included in the study. All groups were followed-up for 5 weeks.

### Body weight and Lee index

All animals were weighed once per week throughout the protocol period. To determine the body mass index, Lee index was calculated by cube root of body weight (g) × 10/naso-anal length (mm) [21, 22].

### Glycemia and triglycerides measurements

At the beginning and at the end of the protocol, all animals underwent 4 h of fasting. A drop of blood from the tail vein was collected to measure plasma glucose using a glucometer, and another drop was collected for triglyceride measurement with Accu-Chek e Accutrend GTC (Hoffman-La Roche Ltd., IN, EUA), respectively.

### Insulin tolerance test (ITT)

ITT was performed at the end of the protocol. Baseline glucose levels were determined according to Furuya et al. 2003. Insulin (0.75 units/kg) was injected subcutaneously. Then, blood samples were collected from the tail vein at 0 (just before the insulin injection), 4, 8, 12 and 16 min (after the insulin injection) for the glucose assay. Plasma glucose was measured from blood samples collected from the tail vein with the use of a glucometer (Accu-Check; Hoffman-La Roche Ltd.) [23–25]. Glycemia values for 4 to 16 min were used to calculate the constant decrease of plasma glucose (kITT) [26].

### Echocardiographic evaluations

Echocardiographic evaluations were blindly performed by an expert and in accordance to the guidelines of the American Society of Echocardiography. Rats were anaesthetized (50 mg/kg ketamine and 12 mg/kg xylazine, intraperitoneal injection) and images were obtained with a 10–14 mHz linear transducer in a SEQUOIA 512 (ACUSON Corporation, Mountain View, CA, USA) for measurements of left ventricular function and morphology. Echocardiographic parameters were measured as previously described in detail elsewhere [27, 28].

### Hemodynamics and baroreflex sensitivity

One day after the echocardiographic evaluation, two polyethylene catheters filled with 0.06 ml of saline were implanted into the femoral artery and femoral vein (PE-10). Rats were anesthetized with isoflurane (1 a 2.5%), for direct measurements of arterial pressure and drug administration, respectively. After this procedure, the animals were allowed to recover in individual cages. The rats were taken to the recording room at least 30 min before the beginning of the experiment, and a quiet environment was maintained to avoid any stress. On the next day, the arterial cannula was connected to a strain-gauge transducer (Blood Pressure XDCR; Kent Scientific, Torrington, CT), and arterial pressure signals and pulse interval (heart rate) were digitally recorded over a 30-min period in conscious animals, moving freely during the experiments using a data acquisition system (WinDaq, 2 kHz, DATAQ, Springfield, OH, USA) as previously described [21, 29]. Beat-by-beat time series of systolic, diastolic, and mean arterial pressures were generated. Heart rate was measured from successive diastolic pulse intervals. This basal acquisition was used to evaluate heart rate variability and systolic arterial pressure variability.

For baroreflex sensitivity evaluation, sequential bolus injections of increasing doses of phenylephrine (0.25 to 32 μg/kg) and sodium nitroprusside (0.05 to 1.6 μg/kg) were given to increases or decreases in mean arterial pressure responses (for each drug), ranging from 5 to 40 mmHg.32. Peak increases or decreases in mean arterial pressure after phenylephrine or sodium nitroprusside injection and the corresponding peak reflex changes in heart rate were recorded for each dose of the drug. A 3–5 min interval between doses was necessary for arterial pressure to return to baseline. Baroreflex sensitivity was expressed as bradycardic response and tachycardic response, expressed as bpm/mmHg, as described elsewhere in beats per minute per millimeter of mercury [27, 29, 30]. After the hemodynamic recordings, rats were euthanized by decapitation. The heart, pancreas and gastrocnemius were rapidly removed, rinsed in ice-cold 0.9% NaCl solution and weighed.

### Heart rate variability

The heart rate variability was performed by linear methods in time and frequency domain by Cardioseries® v2.4. Temporal series of pulse interval and systolic blood pressure from baseline recordings were analyzed and total heart rate variability (HRV) and systolic arterial pressure variability (SAPV) were calculated. The mean square root of differences between consecutives PI (RMSSD), an index of parasympathetic modulation in time domain, was also evaluated. For frequency domain, the interpolated wave of these same basal periods were divided in segments of 512 beats with overlap of 50% and processed by Fast Fourier Transform. One spectrum was obtained by each segment and the oscillatory components were quantified in low frequency (LF; 0.20 to 0.75 Hz), which indicates sympathetic modulation predominance, and high frequency (HF, 0.75 to 3.00 Hz), which indicates parasympathetic modulation predominance [31].

### Statistical analysis

All data are expressed as mean ± SEM. The two-way analysis of variance followed by Newman-Keuls post hoc test was used to compare groups. Repeated measures two-way analysis of variance was used when necessary. Pearson’s correlation was used for the study of associations between variables. Kaplan-Meier method was used to calculate survival rate and curves were compared by logrank test. Statistical calculations were performed using Graph Pad Prism version 6.0. Significance level was established at *p* < 0.05.

## Results

### Body weight, Lee index and tissue weight

**Fig. 1:**
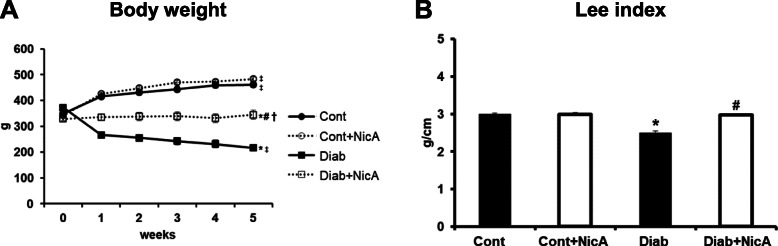
1A, Body weight of control (Cont, *n* = 8); control nicotinamide (Cont+NicA, *n* = 7); diabetes (Diab, n = 7) and diabetes nicotinamide (Diab+NicA, *n* = 8) groups; *p ≤ 0.0001 vs. Cont; ^#^*p* ≤ 0.0001 vs. Diab; ^†^*p* ≤ 0.0001 vs. Cont+NicA; ^‡^*p* ≤ 0.0001 week 5 vs. week 0 in the same group. 1B, Lee index of control rats (Cont, *n* = 8); control nicotinamide (Cont+NicA, *n* = 7); diabetes (Diab, *n* = 7) and diabetes nicotinamide (Diab+NicA, *n* = 8); *p ≤ 0.0001 vs. Cont; ^#^*p* ≤ 0.0001 vs. Diab

At the end of the protocol Cont and Cont+NicA groups presented body weight gain in relation to initial values. In contrast, Diab group presented body weight loss throughout the protocol, and nicotinamide prevented body weight loss in diabetic rats (Fig. 1A). Moreover, diabetic rats presented decreased Lee index when compared to other groups, and nicotinamide in diabetic rats reestablished this parameter (Fig. 1B). Both diabetic groups presented reduction in pancreas weight in relation to non-diabetic rats (*p* < 0.05) (Cont:1.48 ± 0.11 g; Cont+NicA:1.86 ± 0.21 g; Diab:1.23 ± 0.06 g; Diab+NicA:1.11 ± 0.07 g). Similarly, gastrocnemius muscle weight was also lower in diabetic groups (Diab and Diab+NicA; 1.16 ± 0.10 g and 1.51 ± 0.11 g, respectively) when compared to controls (Cont and Cont+NicA; 2.29 ± 0.19 and 2.04 ± 0.26 g, respectively). Absolute cardiac mass was not different among groups (Cont:1.50 ± 0.05 g; Cont+NicA:1.72 ± 0.08 g; Diab:1.44 ± 0.09 g; Diab+NicA:1.34 ± 0.07 g).

### Metabolic parameters

**Fig. 2:**
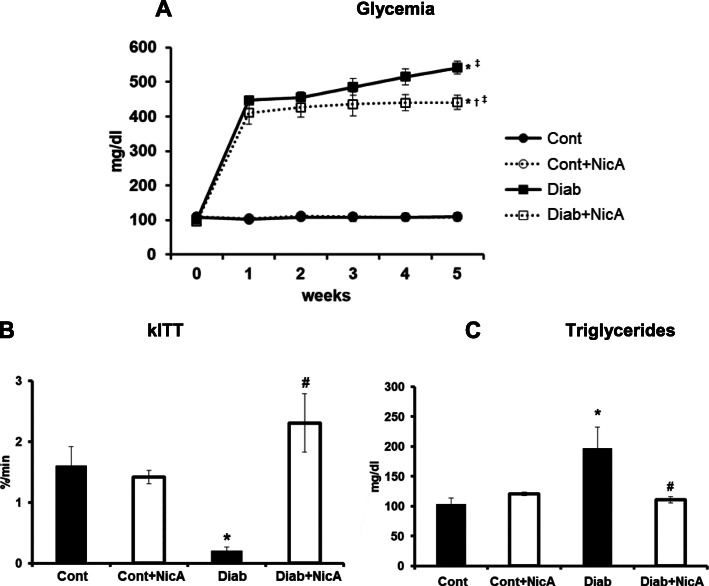
2A, Fasting glycemia of control (Cont, *n* = 8); control nicotinamide (Cont+NicA, *n* = 7); diabetes (Diab, *n* = 7) and diabetes nicotinamide (Diab+NicA, *n* = 8) groups; *p ≤ 0.0001 vs. Cont; ^†^*p* ≤ 0.0001 vs. Cont+NicA; ^‡^*p* ≤ 0.0001 week 5 vs. week 0 in the same group. 2B, Insulin tolerance test evaluated by the constant decrease for plasma glucose (kITT) of control rats (Cont, *n* = 8) control nicotinamide (Cont+NicA, *n* = 7); diabetes (Diab, *n* = 7) and diabetes nicotinamide (Diab+NicA, *n* = 8); *p ≤ 0.0001 vs. Cont; ^#^*p* ≤ 0.0001 vs. Diab. 2C, Fasting triglycerides levels of control rats (Cont, *n* = 8); control nicotinamide (Cont+NicA, *n* = 7); diabetes (Diab, *n* = 7) and diabetes nicotinamide (Diab+NicA, *n* = 8); *p ≤ 0.05 vs. Cont; ^#^*p* ≤ 0.01 vs. Diab

Before induction, all groups had similar glycemia values (week 0) (Fig. 2A). As expected, STZ induced a sharp increase in glycemia (Diab: 541.28 ± 18.68 mg/dl) when compared to non-diabetic rats, and although nicotinamide reduced glycemia in STZ rats (440.87 ± 20.96 mg/dl), these values were still higher than Cont and Cont+NicA groups (110.00 ± 3.48 and 108.50 ± 1.52 mg/dl, respectively). Concomitantly, Diab rats presented an important deficiency in the insulin tolerance test (0.21 ± 0.05 mg/dl/min), which was totally prevented by nicotinamide, as observed in Diab+NicA group (2.30 ± 0.47 mg/dl/min) (Fig. 2B). STZ also induced an increase in triglycerides levels (180.42 ± 34.70 mg/dl), while nicotinamide in STZ-induced rats attenuated this increase (111.00 ± 5.12 mg/dl) (Fig. 2C).

### Hemodynamics and cardiovascular autonomic modulation assessments

Results from hemodynamic and cardiovascular autonomic modulation are presented in Table 1. Diab rats presented resting bradycardia and decreased mean blood pressure levels when compared to control groups, while Diab+NicA rats presented heart rate and blood pressure values equivalent to non-diabetic groups. Heart rate and blood pressure variabilities were not different among groups. Despite the absence of significant alterations in the LF component, which represents sympathetic predominance in autonomic modulation, Diab group presented reduced absolute HF component, indicating parasympathetic impairment. Positive effect of nicotinamide in diabetic rats was observed in parameters of parasympathetic modulation, as absolute HF component and RMSSD.

**Table 1:** Hemodynamics and cardiovascular autonomic modulation in control (Cont, *n* = 8), control nicotinamide (Cont+NicA, *n* = 7), diabetic (Diab, *n* = 7), and diabetic nicotinamide (Diab+NicA, *n* = 8) groups

	Cont	Cont + NicA	Diab	Diab + NicA	***P*** value
**Heart rate (bpm)**	338 ± 6	343 ± 6	255 ± 6*^†^	299 ± 10^#^	*p* < 0.05
**Mean blood pressure (mmHg)**	110 ± 2	115 ± 2	94 ± 3^*†^	101 ± 2^#^	*p* < 0.05
**HRV (ms**^**2**^**)**	105.1 ± 15.5	101.4 ± 17.6	129.6 ± 28.9	120.9 ± 17.7	*p* > 0.05
**RMSSD (ms)**	4.8 ± 0.3	6.7 ± 0.6	5.4 ± 0.2	9.3 ± 1.2^*#^	*p* < 0.05
**LF (ms**^**2**^**)**	4.5 ± 0.7	4.2 ± 1.0	3.6 ± 0.8	5.2 ± 0.7	*p* > 0.05
**HF (ms**^**2**^**)**	13.0 ± 0.8	13.8 ± 2.2	7.0 ± 1.2^*†^	13.7 ± 1.6^#^	*p* < 0.05
**LF (n.u.)**	22.8 ± 2.8	19.8 ± 3.3	25.6 ± 3.7	23.5 ± 2.4	*p* > 0.05
**HF (n.u.)**	77.1 ± 2.8	80.1 ± 3.3	71.2 ± 3.7	76.5 ± 2.4	*p* > 0.05
**LF/HF**	0.30 ± 0.04	0.30 ± 0.05	0.46 ± 0.04	0.36 ± 0.04	*p* > 0.05
**SAPV (mmHg**^**2**^**)**	43.9 ± 1.8	42.2 ± 4.8	21.8 ± 10.0	33.1 ± 3.8	*p* > 0.05

*HRV* Heart rate variability; *SAPV* systolic arterial pressure variability computed from 0.20 to 3 Hz (total power), low-frequency (LF: 0.20–0.75 Hz) and high-frequency (HF: 0.75–3 Hz) bands; *RMSSD* root mean square of successive differences; *n.u* normalized units. *p < 0.05 vs. Cont; ^#^*p* < 0.05 vs. Diab, ^†^*p* < 0.05 vs. Cont+NicA

### Baroreflex

**Fig. 3:**
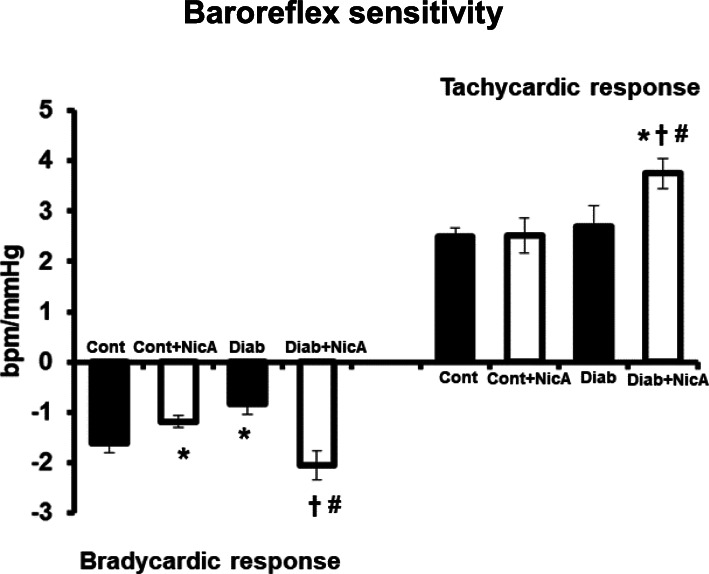
Baroreceptor reflex sensitivity evaluated by bradicardiac and tachycardic responses of control (Cont, *n* = 8); control nicotinamide (Cont+NicA, *n* = 7); diabetes (Diab, *n* = 7) and diabetes nicotinamide (Diab+NicA, n = 8) groups; *p ≤ 0.05 vs. Cont; ^#^*p* ≤ 0.05 vs. Diab; ^†^*p* ≤ 0.05 vs. Cont+NicA

Results of baroreflex sensitivity evaluated by heart rate changes to vasoactive drugs revealed a reflex impairment of the bradycardic response in Diab vs. Cont. nicotinamide treatment in STZ rats increased both tachycardic and bradycardic responses in relation to Diab (Fig. 3).

### Cardiac morphometry and function

Echocardiography analysis results are shown in Table 2. Left ventricular diameter during systole was not different among groups; however, left ventricular diameter in diastole was diminished in Diab rats vs. Cont. Diabetes did not interfere in interventricular septum thickness in diastole, in posterior wall in diastole, and in relative wall thickness. Functional left ventricular analysis revealed diastolic dysfunction with preserved ejection fraction in Diab rats. Isovolumetric relaxation time was higher in Diab group in relation to Cont group, and Diab+NicA rats showed lower values than Diab. The ratio between E wave (early diastolic transmitral flow velocity) and isovolumetric relaxation time (E/IVRT), which was reduced in Diab group, was similar to Cont in Diab+NicA group. No differences in systolic function parameters were observed among groups.

**Table 2:** Echocardiographic measurements in the left ventricle of (Cont, *n* = 8), control nicotinamide (Cont+NicA, *n* = 7), diabetic (Diab, *n* = 7), and diabetic nicotinamide (Diab+NicA, *n* = 8) groups

	Cont	Cont + NicA	Diab	Diab + NicA	***P*** value
**LVSD (cm)**	0.54 ± 0.02	0.56 ± 0.01	0.46 ± 0.02	0.51 ± 0.01	*p* > 0.05
**LVDD (cm)**	0.87 ± 0.02	0.87 ± 0.01	0.77 ± 0.02*	0.83 ± 0.02	*p* < 0.05
**IVSD (cm)**	0.147 ± 0.03	0.152 ± 0.002	0.145 ± 0.006	0.125 ± 0.003	*p* > 0.05
**PWD (cm)**	0.150 ± 0.003	0.158 ± 0.003	0.148 ± 0.06	0.126 ± 0.003	*p* > 0.05
**RWT (cm)**	0.35 ± 0.02	0.36 ± 0.01	0.39 ± 0.02	0.31 ± 0.01	*p* > 0.05
**EF (%)**	76 ± 1	74 ± 1	79 ± 2	76 ± 2	*p* > 0.05
**FS (%)**	46.2 ± 7.7	44.6 ± 8.6	40.6 ± 1.6	45.9 ± 7.9	*p* > 0.05
**IVRT (ms)**	33.2 ± 0.8	29.4 ± 1.2	45.1 ± 1.8*	36 ± 1.6^#^	*p* < 0.05
**E’ (ms)**	0.046 ± 0.003	0.045 ± 0.002	0.044 ± 0.001	0.042 ± 0.002	*p* > 0.05
**A’ (ms)**	0.037 ± 0.004	0.031 ± 0.001	0.048 ± 0.004	0.051 ± 0.004	*p* > 0.05
**E/IVRT**	0.021 ± 0.002	0.028 ± 0.002	0.013 ± 0.001*	0.018 ± 0.001^#^	*p* < 0.05

*LVSD* left ventricle diameter in systole; *LVDD* left ventricle diameter in diastole; *IVSD* interventricular septum in diastole; *PWD* posterior wall in diastole; *RWT* relative wall thickness; *EF* ejection fraction; *FS* fractional shortening; *IVRT* isovolumetric relaxation time; *E’* early diastolic mitral annular tissue velocity; *A’* late atrial diastolic mitral annular velocity; *E/IVRT* ratio between early diastolic transmitral flow velocity and isovolumetric relaxation time. *p < 0.05 vs. Cont; ^#^*p* < 0.05 vs. Diab

### Survival curve

**Fig. 4:**
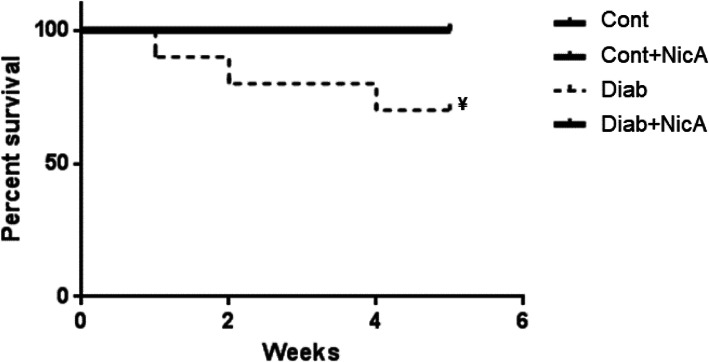
Survival percentage estimated by the Kaplan-Meier method of control (Cont, *n* = 8); control nicotinamide (Cont+NicA, *n* = 7); diabetes (Diab, *n* = 7) and diabetes nicotinamide (Diab+NicA, *n* = 8) groups; ^¥^ ≤ 0.05 vs. all groups

Mortality among studied rats was evaluated for 5 weeks. Our results showed that nicotinamide was an effective intervention to increase survival rate in STZ-induced diabetic rats. Figure 4 depicts the survival curve for all studied groups.

### Correlation analysis

Pearson’s correlation analysis using all diabetic rats showed strong associations between the index of reflex bradycardia and fasting glycemia (r = 0.75, *p* = 0.003), and the rate constant for plasma glucose disappearance (kITT) (r = − 0.75, p = 0.003). In addition, RMSSD was also associated with kITT (r = 0.61, *p* = 0.04). Moreover, body weight gain was associated with both bradycardia and tachycardia indexes with (r = 0.66, *p* = 0.013; r = 0.59, *p* = 0.03, respectively), as well as with kITT (r = 0.78, *p* = 0.001) and RMSSD (r = 0.58, *p* = 0.03).

## Discussion

Our results confirmed that a single dose of nicotinamide before diabetes induction by STZ can attenuate most of the severe alterations observed in this diabetes model. The main novelty reported by the present study is that this protective effect of nicotinamide was associated with preserved baroreflex sensitivity and parasympathetic modulation. The improvement in these autonomic parameters reflected in better metabolic profile, as observed in the correlation analyses results, and increased survival rate in diabetic rats that received nicotinamide when compared to rats that received only STZ.

It is well known that STZ is a potent cytotoxic drug to pancreatic beta-cells in rats. Briefly, STZ causes DNA damage of insulin-secreting cells, and this injury leads to mechanisms of DNA repair, mitochondria dysfunction and ATP depletion [32, 33]. As a result, rats present severe diabetes symptoms, similar to what was observed in our STZ animals, as exacerbated levels of glycemia and body weight loss. On the other hand, nicotinamide protects pancreatic beta-cells from DNA damage; therefore, it blunts the development of several STZ-induced diabetes characteristics, as increased hyperglycemia and body weight loss.

In fact, hyperglycemia above 400 mg/dl is usually observed in STZ-induced diabetic rats [20, 34–36] and it is associated with high mortality [29, 37]. However, in the present study, nicotinamide-induced protective effects seemed to be independent of glycemia levels, as Diab+NicA rats still showed high glycemia values (~ 400 mg/dl) despite being lower than Diab rats. Therefore, we investigated other factors which could mediate the improvements observed in Diab+NicA rats.

It is well known that autonomic dysfunction is in the genesis and progression of several diseases. Parasympathetic dysfunction was associated with insulin resistance in fructose-fed rats [23]. Indeed, Diab rats presented insulin resistance evidenced by rate constant for insulin tolerance test (kITT), and this parameter was totally normalized in Diab+NicA. Moreover, parasympathetic dysfunction and reflex mechanisms impairments in STZ-induced diabetic rats have already been reported [20, 34, 37, 38]. This impairment may be a result of alterations in both the efferent limb of the reflex arc, and the central nervous system [39].

Baroreflex sensitivity impairment in the STZ model of diabetes has also been described [15, 29, 30, 37]. In our study, only the bradycardic response was impaired. This corroborates with the autonomic modulation profile of the studied rats, as in diabetic rats parasympathetic modulation was reduced. Even though Diab+NicA rats were still hyperglycemic, glycemia was about 20% lower than Diab rats. De Angelis et al. (2002) demonstrated that a better glycemic control is related to baroreflex sensitivity and autonomic improvements [39].

Accordingly, we found important associations between insulin resistance evaluated by kITT and RMSSD, a time-domain index of cardiac parasympathetic modulation. Also, kITT was associated with the index of reflex bradycardia, showing the interaction between insulin resistance and reflex control of blood pressure. Mechanisms underlying this association are not fully understood; however, some studies have demonstrated that improvements in cardiac oxidative stress profile may contribute to an optimal baroreflex sensitivity and insulin action in aged rats [40, 41].

STZ-induced diabetic rats also presented hemodynamic alterations which are closely related to autonomic dysfunction. Resting bradycardia and blood pressure decrease is a frequent observation [29, 37, 38, 42]. Mostarda et al. (2009) showed that resting bradycardia in diabetic rats occurs due to decreased intrinsic heart rate, while decreased blood pressure is linked to a reduction in cardiac output [29].

Cardiac function in STZ-induced diabetic may also be impaired, as systolic and diastolic dysfunctions have been described [28, 43]. Indeed, Diab rats had decreased IVRT and E/IVRT ratio, indicating diastolic dysfunction. We did not find systolic dysfunction or expressive morphological abnormalities; however, our follow-up period was shorter than other studies.

Diabetes patients present high levels of mortality mainly due to cardiovascular complications [1]. In asymptomatic individuals, it was observed that up to 20% of them presented impairment of cardiovascular autonomic function [44]. This information highlights the importance of neuropathy in the time course of diabetic disease.

Another characteristic of diabetes is body weight loss. In our study, rats that received nicotinamide were able to maintain body weight stable throughout the studied period, while, as expected, diabetic rats had a severe weight loss (> 40%). Guo et al. (2019) reported that nicotinamide had a protective effect on skeletal muscle atrophy in STZ-induced diabetic mice, which may be through inhibition of TGF-b1/Smad2 pathway [45]. Preservation of body mass seems to be an important feature in diabetes. The correlation analyses showed that diabetic rats with preserved body weight mass presented increased baroreflex sensitivity, increased RMSSD, and decreased insulin resistance.

Altogether, the improvements induced by nicotinamide in diabetic rats resulted in increased survival rate. In 13 weeks, survival in STZ-induced diabetic rats was approximately 50% [29]. In our study, Diab rats survival rate in 5 weeks was 70%, while Diab+NicA rats presented 100% survival rate in the same period. Indeed, nicotinamide in addition to intensive insulin therapy during 2 years after type 1 DM diagnosis may improve metabolic control [46]. In contrast, 12 weeks supplementation of dietary nicotinamide riboside did not improve insulin sensitivity and other metabolic parameters in obese insulin resistance men [47]. Our new data showing improvement of autonomic modulation and in mortality in nicotinamide-treated animals may indicate a protective action that should be tested in long-term studies.

Diabetes induced by STZ and nicotinamide remains stable for a long time and thus this model of diabetes is suitable not only for short-term but also for long-term studies. Moreover, this model is very useful in investigations of different aspects of diabetes, including diabetic complications and anti-diabetic properties of new drugs and natural compounds [48].

## Conclusion

Nicotinamide associated with STZ is a more stable model of experimental diabetes by preventing STZ-induced baroreflex impairment, parasympathetic dysfunction and insulin resistance in rats, independently of glycemia levels. In association with improved metabolic profile, and preserved body weight mass, these protective effects of nicotinamide resulted in increased survival rate.

## Data Availability

Not applicable.
